# Google Translate Voice App vs. Qualified Interpreters: An Exploratory Study of Clinical Accuracy in Real-World Speech/Voice Encounters

**DOI:** 10.3390/healthcare14162497

**Published:** 2026-08-11

**Authors:** Iris Feinberg, Heewon Lee-Laminack, Elizabeth L. Tighe, Ifedola Owoeye

**Affiliations:** 1 Adult Literacy Research Center, Department of Learning Sciences, Georgia State University, Atlanta, GA 30303, USA; heewonlee9304@gmail.com (H.L.-L.); iowoeye1@student.gsu.edu (I.O.); 2 Department of Psychology, Georgia State University, Atlanta, GA 30303, USA; etighe@gsu.edu

**Keywords:** qualified medical interpreter, AI-voice assisted app, interpretation, limited English proficiency, language barriers

## Abstract

**Highlights:**

**What are the main findings?**

- Using real clinical speech segments, Google Translate in speech/voice mode had significantly higher linguistic and clinical error rates than qualified medical interpreters across six languages (33.3% vs. 4.8%).
- Error rates were worse for less commonly spoken languages, amplifying equity concerns.

**What are the implications of the main findings?**

- Qualified in-person medical interpreters provide more accurate verbal translations than Google Translate in speech/voice mode using real world clinic speech segments that include discourse markers, fillers, hesitation markers, and descriptive language.
- AI apps like Google Translate in speech/voice mode that are frequently used in low-resourced clinical settings may not provide accurate interpretation that reflects how people really speak and require further study.

**Abstract:**

**Background/Objectives**: AI voice interpretation applications like Google Translate in speech/voice mode are increasingly used in clinical settings to address verbal language access challenges, yet evidence comparing their performance to qualified in-person medical interpreters in authentic clinical encounters remains limited. The aim of this study is to compare the linguistic and clinical accuracy of AI-based Google Translate in speech/voice mode and qualified in-person medical interpretation using spoken real-world speech segments from clinical encounters. **Methods**: Outpatient clinical encounters involving patients with limited English proficiency were audio-recorded. Fourteen physician speech segments (mean length 78.5 words) representing common diagnostic, treatment, and counseling content were extracted, spoken in English into Google Translate speech/voice mode, and translated into six languages. Audio recordings of the same sentence segments were provided to qualified in-person medical interpreters. All non-English translations were back-translated into English by professional interpreters. Researchers and a clinician evaluated the back-translated English speech segments for linguistic accuracy, completeness, and clinical significance. Qualitative analyses examined error patterns and contextual loss; quantitative comparisons assessed error rate differences across languages and interpretation conditions. **Results**: Google Translate in speech/voice mode exhibited significantly higher linguistic errors (*χ*^2^[1] = 19.78, *p* < 0.001) and clinical accuracy errors (*χ*^2^[1] = 45.07, *p* < 0.001) than qualified medical interpreter translations. Clinically significant error rates were 33.3% for Google Translate in speech/voice mode versus 4.8% for interpreter-generated translations. Error rates were also higher for less commonly spoken languages compared to commonly spoken languages when using Google Translate in speech/voice mode (42.9% vs. 14.3%). **Conclusions**: Qualified in-person interpreters provided translated speech segments that had fewer errors that were either linguistically or clinically significant, and remain essential for safe, accurate clinical communication.

## 1. Introduction

Language barriers remain a persistent and well-documented challenge in healthcare systems worldwide for patients with limited English proficiency (LEP). Inadequate communication between clinicians and patients with LEP is consistently associated with lower comprehension of diagnoses and treatment plans, reduced adherence to medical recommendations, increased risk of medical error, and poorer overall health outcomes [1,2,3]. Communication difficulties can compromise shared decision-making, limit patients’ ability to express symptoms accurately, and undermine trust in healthcare providers. As healthcare delivery becomes increasingly complex and technologically mediated, the consequences of ineffective communication for LEP populations have become more pronounced, reinforcing language access as a core component of healthcare quality and equity. Recent studies further indicate that language discordance contributes to disparities in hospitalization rates, emergency department utilization, and patient-reported experiences of care [4,5,6,7]. These patterns highlight the central role of language access in advancing health outcomes and health equity.

Qualified medical interpreters have been shown to substantially improve communication quality, patient safety, and patient satisfaction in clinical encounters involving patients with LEP [4]. Interpreters facilitate accurate information exchange, promote patient engagement, and reduce misunderstandings that can lead to confusion about treatment or delayed care. Empirical studies indicate that interpreter-mediated visits are associated with higher rates of preventive service use, better chronic disease management, and improved adherence to prescribed therapies [1]. Despite this evidence, access to qualified interpreters remains uneven across healthcare settings. Rural hospitals, community clinics, and safety-net systems frequently report limited interpreter availability, particularly for languages spoken by smaller immigrant or refugee populations. Structural constraints—including financial limitations, workforce shortages, scheduling challenges, and inadequate reimbursement mechanisms—continue to hinder consistent provision of interpreter services [1]. Workforce pipeline challenges and limited certification infrastructure further constrain interpreter availability in underserved regions [8,9]. These systemic barriers create persistent gaps that perpetuate reliance on ad hoc or informal interpretation practices that compromise quality and safety.

In response to these persistent gaps in language access, digital and artificial intelligence (AI)–based language technologies have increasingly been introduced into clinical environments. AI voice assisted apps and interpretation systems, mobile translation applications, and machine translation platforms offer rapid, on-demand access to multilingual communication support and are often promoted as scalable and cost-efficient alternatives to traditional interpreter services [10]. These technologies have been adopted in outpatient clinics, emergency departments, inpatient units, and telehealth settings, particularly in resource-constrained environments. Google Translate is considered the most widely used voice-assisted app for cross-cultural communication because it is a free and ubiquitously used platform and allows for voice translation in over 200 widely spoken languages, representing over 614 million users. For free/volunteer healthcare settings with limited budgets, the cost and accessibility of Google Translate make it the dominant AI tool for voice and text translation throughout the world despite documented challenges with translation accuracy [11,12].

Existing research on technology-assisted interpretation has produced mixed findings. Studies evaluating machine translation and mobile translation applications suggest that these tools can achieve acceptable performance for simple or standardized content in highly used languages, such as Spanish and Vietnamese [13,14,15,16,17]. For routine instructions, appointment scheduling, or basic symptom descriptions, automated systems may provide reasonably accurate translations. However, accuracy declines substantially for complex medical terminology, nuanced counseling, emotionally sensitive discussions, and culturally embedded communication [10]. Subtle shifts in tone, modality, or contextual meaning may be lost, potentially altering clinical intent. These limitations may restrict the appropriateness of automated tools in high-stakes or ethically sensitive encounters. Importantly, the majority of studies in this literature have evaluated machine translation of written text rather than spoken clinical communication—most commonly written discharge instructions assessed for accuracy by bilingual reviewers [13,18]. The more limited body of research examining voice-to-voice machine translation in live clinical encounters has focused primarily on user satisfaction and goal achievement rather than independent verification of translation accuracy, leaving the linguistic and clinical error rates of AI voice interpretation in spoken medical discourse largely unexamined [19].

Performance disparities are particularly pronounced for less commonly spoken languages, reflecting limited training data and algorithmic bias within AI systems [20]. Languages with fewer digital resources such as Amharic, Arabic, Burmese, and Swahili are often underrepresented in training datasets, leading to higher error rates and inconsistent output quality. These limitations raise serious concerns about the potential for clinically meaningful mistranslations, omissions, and distortions when AI tools are used without oversight [13]. Even minor translation errors can have significant consequences in contexts involving medication dosing, informed consent, or discharge planning. Studies have shown that error rates increase substantially for indigenous, refugee, and regional languages [21]. Such disparities risk reinforcing existing structural inequities in healthcare delivery [22,23].

Despite growing reliance on AI-based interpretation, direct comparisons between these tools and qualified in-person interpreters in real-world clinical encounters remain limited [10,24]. Much of the existing literature relies on simulated interactions, written translation tasks, or narrowly defined clinical scenarios [1]. Such designs may not adequately capture the complexity of authentic patient–provider communication. As a result, current evidence may overestimate real-world performance [1,25]. This study explores translation accuracy, including both linguistic accuracy and clinical significance of translation errors of Google Translate AI-based voice interpretation and qualified in-person medical interpretation using 14 recorded sentence segments from real-world clinical encounters in six languages (Amharic, Arabic, Burmese, Spanish, Swahili, Vietnamese) which we grouped as common (Spanish and Vietnamese) and less common (Amharic, Arabic, Burmese, Swahili). Specifically, we sought to describe not only translation accuracy between qualified translators and Google Translate AI-assisted voice interpretation but to determine whether the errors displayed clinical significance that could impact patient care and health outcomes.

Clinical communication is not simply the transmission of propositional content—it is a richly layered pragmatic event shaped by context, speaker intent, and social relationship. Real spoken language involves discourse features that extend well beyond lexical meaning, including prosody, hedging, politeness strategies, implicature, and the sequential organization of conversational turns. Clinicians routinely use indirect speech acts, softening language, and culturally embedded expressions when delivering diagnoses or counseling patients about treatment—communicative choices that carry significant pragmatic force and that research has shown are central to effective, patient-centered care [26]. These features are not incidental to clinical talk; they shape how patients understand their condition, assess risk, and build trust with their provider. AI voice interpretation applications like Google Translate are built on neural machine translation (NMT) architectures that operate primarily at the sentence level, optimizing for surface-level linguistic form rather than discourse-level meaning [27]. As a result, NMT systems have well-documented difficulty with discourse phenomena such as pronoun resolution, politeness registers, and conversational implicature—features that require broader contextual and interactional knowledge to render accurately [27,28]. When these subtle but consequential pragmatic dimensions of clinical speech are lost or distorted in translation, the output may be technically intelligible but communicatively misleading, with potential consequences for patient understanding, safety, and trust [29].

To address these aims, this study examined translation accuracy across both modality and language grouping. First, it compared Google Translate in speech/voice mode (APP) and qualified medical interpreter (QMI) translations in terms of overall error rates. We chose Google Translate because it is dominantly used by healthcare providers in free/volunteer clinics that serve refugees and immigrants [1]. We used real-world physician speech segments which include pause fillers including “ah” and “um” because that is how people speak in the real-world and it is how their speech segments would need to be translated by any voice-assisted AI app. Although not prescriptively correct English, real-world speech is functional and communicative, and it is what needs to be translated for patients. Second, it examined whether error patterns differ between common and less common languages within each translation condition. Finally, it assessed the likelihood of clinically accurate translations across language groups for both APP and QMI conditions. The research questions for this study are:

1. Is there a significant difference in linguistic or clinical error rates between APP and QMI translations when translating real-world physician speech?
2. Within each condition, is there a significant difference in linguistic or clinical error rates between common and less common languages?
3. What are the odds of APP translations being clinically accurate in common versus less common languages?
4. What are the odds of QMI translations being clinically accurate in common versus less common languages?

## 2. Materials and Methods

### 2.1. Sample

Fourteen English-speaking physician speech segments were sampled from an existing corpus of audio-recorded outpatient clinical encounters [30]. Selected segments represented common diagnostic, treatment, and counseling content and contained medically relevant terminology. Sentences were chosen to reflect realistic patient-facing medical comments and instructions in outpatient settings. The mean length was 78.5 words per segment (range: 33–140 words), representing a range of common clinical communication contexts including diagnostic explanation, treatment planning, and patient counseling. See Table 1 for a sample of selected speech segments. All source audio recordings were de-identified. No patient-identifying information was included in translated materials. The study protocol adhered to ethical standards for secondary data analysis of clinical communication.

### 2.2. Study Design

This study employed a comparative accuracy design to evaluate translation accuracy and clinical accuracy of speech segments translated by Google Translate speech/voice mode (APP) and by qualified medical interpreters (QMIs). Google Translate speech/voice mode was selected because it includes all our study languages and is a commonly used tool in clinical settings [31]. Speech segments were provided in audio format. Google Translate is primarily based on Neural Machine Translation (NMT), specifically using Transformer-based deep learning models, enhanced by Large Language Models (LLMs) and Generative AI components for context and naturalness in speaking [32]. The sentences were translated in early 2026, using the most up-to-date commercially available version of Google Translate. QMIs were qualified by being verified as bilingually fluent and completing one of two 4-week courses (Breaking Boundaries in Healthcare or Bridging the Gap). Upon certification, they also worked as QMIs in our local community. Both training programs are widely recognized as high quality medical interpreter programs including foundational training in ethics, terminology, and interpreting techniques. QMIs completed their interpreter certification training between 2024 and 2025. None of the QMIs utilized in the study are certified by the National Board of Certified Medical Interpreters.

A within-item paired design was used so that each back-translated English sentence served as its own control across translation conditions. Each sentence was verbally translated into six target languages (Amharic, Arabic, Burmese, Spanish, Swahili, and Vietnamese) and then back translated into English. These languages were selected based on prevalence within local clinical populations and availability of local QMIs.

### 2.3. Measures

In the first step, each of the 14 audio sentences was translated under two primary conditions:

- Condition 1: Google Translate speech/voice mode (App Condition)

A study investigator read the English sentences into an iPhone using the Google Translate app. Each sentence was read one time by the same investigator for each language, that is fourteen sentences were read six times. The translated output in the target language was recorded verbatim as an in-language audio file. Each language had 14 sentences translated.

- Condition 2: Qualified Medical Interpreters (QMI Condition)

A study investigator provided the originally recorded English sentences to in-person qualified medical interpreters (QMI) fluent in the respective target language; each QMI translated the same 14 sentences and provided in-language audio files. Each language had 14 sentences translated. Interpreters were credentialed by Breaking Boundaries in Healthcare or Bridging the Gap and experienced in clinical medical interpretation.

In the second step, for both conditions, to assess translation accuracy while minimizing rater bias from direct source comparison, each translated sentence (App and QMI conditions) underwent blind back-translation into English by professional translators who had not participated in the original translation. Back-translators were blinded to the original English sentence. These back translations were recorded as verbal audio files. The audio files were then transcribed verbatim for each language. The total sample size was 14 sentence segments in each of 6 languages in 2 conditions (APP and QMI). The back translated English versions were the unit of measure.

**Figure 1:**
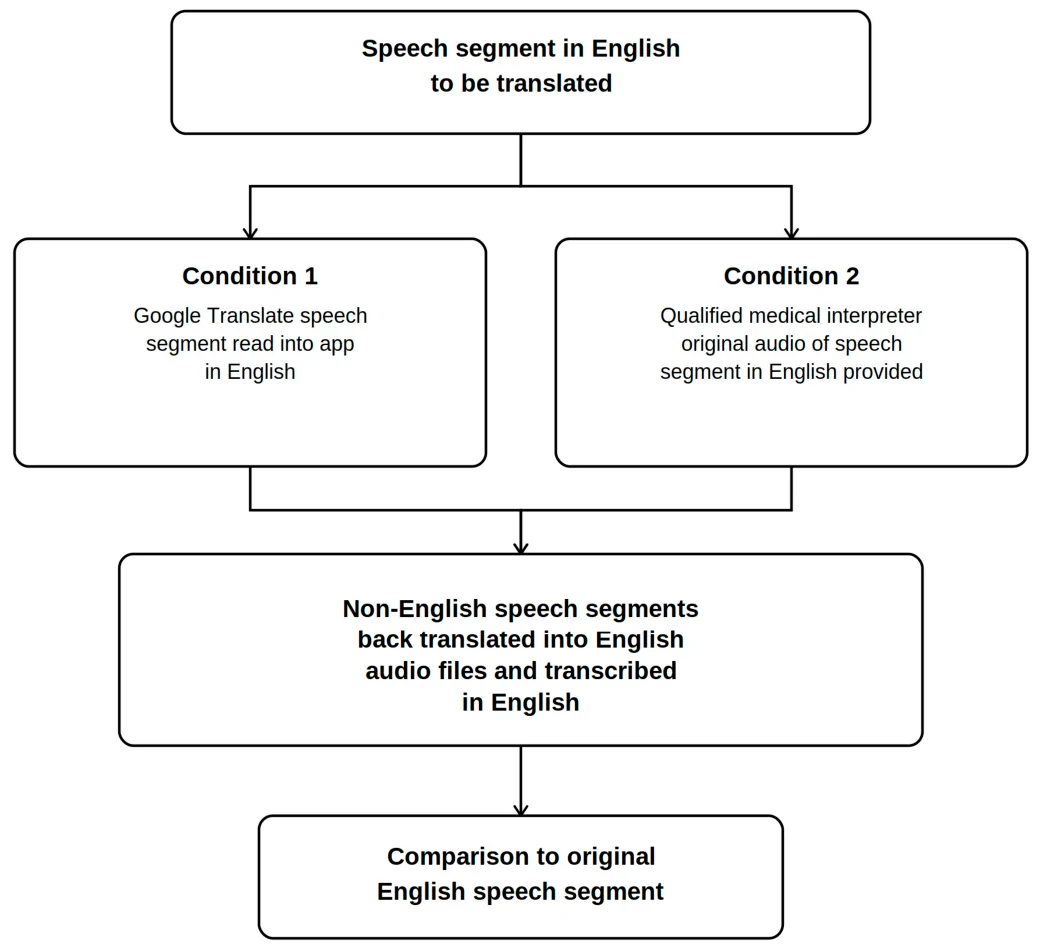
Process for back-translation validation of English speech segments.

In the third step, back-translated English speech segments for each language and in both conditions (APP and QMI) were systematically compared with the original English source sentences for linguistic accuracy, that is, the words were translated exactly as spoken. Two researchers independently reviewed each back-translated segment alongside its original English counterpart. See Figure 1.

Using a predefined coding guide with operational definitions and examples, each segment was coded as:

1. Correct (translated exactly as spoken)
2. Partially wrong (minor inaccuracies, omissions, or distortions that did not fundamentally alter clinical meaning such as saying “how are you” instead of “how are you feeling”)
3. Totally wrong (substantive inaccuracies, omissions, or distortions such as “cyst” instead of “sciatica”)

Two researchers independently coded 20 randomly selected sentence segments to ascertain fidelity to coding and interrater reliability. This represented 12% of the full sample. The study PI reviewed the 20 sentence segments and resolved four discrepancies. A Cohen’s kappa calculation indicated strong agreement between the raters (k = 0.85, *p* < 0.001). Each researcher then independently reviewed either QMI or APP condition for each back-translated segment in comparison to the original source English sentence segment.

Finally, in the fourth step, a clinician with 25 years of clinical practice experience reviewed the partially or totally wrong sentence segments to assess potential impact on clinical interpretation, patient understanding, or patient safety, comparing original English to back-translated English sentence segments for both APP and QMI conditions. The clinician was blinded to condition and language. The clinician evaluated whether discrepancies identified as partially wrong or totally wrong met the threshold for clinical significance. Specifically, segments were coded as:

1 = clinically wrong (presence of medically meaningful inaccuracy, distortion, omission, or unsafe instruction such as “cyst” instead of “sciatica”)

0 = not clinically wrong such as “laceration” and “fissure” which are both types of skin tears and did not clinically change the meaning of the sentence.

Some coding examples are in Table 2.

## 3. Results

All tabulated error rates are presented in Table 3 and separated by translation condition (APP and QMI), language (individual and by common and less common groups), and linguistic and clinical error rates. Below we describe the analyses that could be computed based on the error rates; please note that low error rates, particularly in the clinically wrong for the QMI conditions, precluded us running some of the analyses or may have led to more unstable estimates even with corrections for low counts. These descriptives in Table 3 are crucial for illustrating differences in error rates between the two translation conditions. The unit of measure is the English back-translated sentence. There were 14 sentences for each language and in each condition (APP and QMI). Observations across language groups were analyzed under an assumption of independence. We note, however, that the reuse of the same source sentences across conditions introduces a potential dependency structure that is not explicitly modeled here.

All comparisons (2 × 2 contingency tables) were done from the Table 3 error rate calculations. We used paired tests (McNemar’s chi-square test) between conditions (APP vs. QMI) because it was identical sentences in the same language for both clinically wrong and linguistically wrong. We used a chi-square test between languages because although we used the same sentence, it was translated across languages which are independent. Fisher’s exact tests were used when error rates are small (<5) for independent language groups (for example, QMI linguistically wrong more common = 3.6%).

To assess linguistic error rates, we used a McNemar’s chi-square test to compare error rates on the same 14 sentences between the APP and QMI conditions. The analysis revealed a significant difference (*χ*^2^[1] = 7.12, *p* = 0.008, *OR* = 4.67, 95% CI: 1.34–16.24), suggesting an overall higher linguistic error rate, increased odds of 4.67 for APP translations (see Table 3 for error rates by translation condition).

We used a chi-square test to compare proportions of linguistic errors among independent language groups (commonly spoken [Spanish, Vietnamese] vs. less commonly spoken [Amharic, Arabic, Burmese, Swahili] within the APP translation. This analysis revealed a non-significant difference in proportions of errors among language groups using APP (*χ*^2^[1] = 2.68, *p* = 0.101, *OR* = 2.37, 95% CI: 0.83–6.78; see Table 3 for all error rates by language group). Linguistic error rates between common and less commonly spoken languages were similar when using the APP. We did not run a statistical significance test comparing language groups within the QMI condition because the error rates were very low for the commonly spoken languages (7.1%).

We used a McNemar’s chi-square test to compare linguistic error rates on the same 14 sentences between APP and human interpreters by language group. For less common languages, the analysis revealed a marginally non-significant difference (*χ*^2^[1] = 3.77, *p* = 0.052, *OR* = 3.33, 95% CI: 0.95–11.75), suggesting that although linguistic errors were more likely to occur for APP translations, this difference was not statistically significant (see Table 3 for all error rates). The odds of APP generating linguistic error rates are 2.27 times more than a QMI for less commonly spoken languages. For common languages, the analysis revealed no significant difference (*χ*^2^[1] = 2.00, *p* = 0.157). Although errors were more common for APP translations, there were minimal discordant pairs as well as an overall low error rate (7.1%) for commonly spoken languages in the QMI condition.

To assess clinical error rates, we used a McNemar’s chi-square test to compare error rates on the same 14 sentences between the APP and QMI conditions for clinical correctness. The analysis revealed a significant difference (*χ*^2^[1] = 24.00, *p* < 0.001), suggesting an overall higher clinical error rate for APP translations. Although significant, caution is warranted given the lower number of error rates in the QMI condition. Specifically, the human interpreter generated 4.8% clinically wrong phrases; the APP generated 33.3% clinically wrong phrases over the same sentence segment sample (Table 3).

We used Fisher’s Exact Test to compare proportions of clinical errors among independent language groups (commonly spoken [Spanish, Vietnamese] vs. less commonly spoken [Amharic, Arabic, Burmese, Swahili]) because of the small (<5) error rates in some of the groups. For APP, there was a significant difference in error rates (*p* = 0.013, *OR* = 4.10, 95% CI: 1.32–12.75), suggesting 4.10 increased odds of errors for the less common language group using APP translation. Although significant, caution is warranted given the wide CI, which can suggest uncertainty in the estimated effect and may also be due to the sample size imbalance among language groups (commonly spoken *n* = 28 sentences; less commonly spoken *n* = 56 sentences; see Table 3 for all error rates by language group).

Overall, QMI’s performance was superior in translation accuracy. Caution should be taken, however, when interpreting findings as these are specific to the QMIs who interpreted for this study and should not be generalized to all interpreters of that language. For example, our Amharic interpreter showed the greatest number of errors in translation which could reflect interpreter variability, local workforce constraints, reviewer subjectivity, back-translation limitations, or another factor unknown to us. This could be true of all QMIs in any situation.

We did not run statistical analysis tests within the QMI condition or comparing language groups between translation conditions because of the very small error rates in the QMI condition (less common = 4.8%; more common = 3.6%). Caution is warranted when interpreting the error rates in Table 3 due to the small sample size and the use of the same 14 sentence segments across conditions (APP vs. QMI) and languages, which introduces non-independence among observations and limits statistical power. To address some of the dependency, we used paired tests (McNemar’s chi-square) when comparing conditions within the same sentence segments (APP vs. QMI). For comparisons involving small cell counts (expected frequencies < 5), we used Fisher’s exact test or did not run inferential tests. We acknowledge that these analyses are exploratory and stability and generalizability of findings may be limited. Future studies should consider larger and more diverse samples that allow inferential tests to consider nested structures.

## 4. Discussion

Language access remains a foundational determinant of healthcare quality, safety, and equity for patients with limited English proficiency. While the sample is small, the findings of this exploratory study provide data from real-world oral clinic communications that reinforce longstanding evidence that communication barriers may contribute to compromised treatment adherence and diminished patient engagement [1,6]. These findings are evaluative, assessing the effectiveness of using Google Translate speech/voice mode in clinical settings by analyzing real-world clinic communication with all linguistic challenges intact. The high prevalence of linguistically incorrect and clinically significant errors observed in Google Translate speech/voice mode translations underscores persistent risks associated with substituting this AI-assisted voice technology for human expertise without human intervention and oversight. These exploratory and evaluative findings align with prior research cautioning against uncritical adoption of digital language technologies in high-stakes medical environments [9]. AI voice apps are still considered immature, especially in healthcare, and should be used with caution as standalone solutions to interpretation needs [25]. Further, existing studies of voice-to-voice machine translation in clinical settings have largely relied on self-reported measures of satisfaction and goal achievement or text-based translations, without independently verifying the accuracy of the translations themselves [12,13,18,19]. The present study addresses this methodological gap by evaluating translation accuracy against a corpus of real clinical speech, offering a more objective basis for assessing the risks of AI voice interpretation.

The comparative results highlight meaningful differences in accuracy between qualified in-person interpreters and the Google Translate speech/voice mode. While interpreter-mediated encounters were not error-free, they demonstrated substantially lower rates of linguistically and clinically significant errors than Google Translate. This pattern is consistent with existing literature documenting the capacity of trained interpreters to preserve clinical intent, manage ambiguity, and facilitate culturally responsive communication [1]. In contrast, the disproportionately higher error rates associated with Google Translate speech/voice mode—particularly across less commonly spoken languages—may reflect ongoing limitations in algorithmic training, contextual understanding, and linguistic representation [24]. Consistent with these limitations, our study found that Google Translate speech/voice mode translations frequently involved omissions, mistranslations of medical terminology and medication names, altered sentence meanings, and, at times, distortions that conveyed unintended or opposite meanings. These findings add to the literature that raises concerns about the potential for automated systems to exacerbate existing language-based inequities in healthcare delivery, particularly when translating real world communication.

Our study evaluates translation accuracy and clinical significance through expert review, but does not directly measure patient outcomes, patient or clinician satisfaction, adverse events, trust, or healthcare utilization. In our study, nearly half of all incorrect translations were judged to have meaningful implications for patient care, with the majority originating from t Google Translate speech/voice mode. Errors affecting medication instructions, symptom descriptions, or treatment planning may result in adverse events, delayed care, or compromised informed consent [33]. Patients with LEP are uniquely vulnerable to these risks, as they often lack alternative mechanisms to verify the accuracy of clinical information. These concerns are not merely theoretical. While studies of written machine translation have documented clinically significant errors in discharge instructions—including potentially life-threatening mistranslations of medication directives [13,18]—the present study demonstrates that similar and potentially greater risks arise in spoken clinical communication, where patients have even less opportunity to detect or correct translation errors than they would with written materials they can review. Furthermore, research on voice-to-voice machine translation in live encounters suggests that clinicians may overestimate communication success, with health professionals reporting goal achievement in over 80% of consultations while expressing satisfaction with the quality of communication in only 54%—a discrepancy that underscores the difficulty of detecting translation failures in real time [19].

Translation errors in clinical encounters involving patients with limited English proficiency (LEP) are not merely linguistic inaccuracies; they may carry substantial clinical significance with measurable implications for patient safety, quality of care, and health outcomes. Empirical studies have demonstrated that human interpretation errors—particularly omissions, additions, substitutions, and distortions—can alter diagnostic information, medication instructions, informed consent discussions, and treatment planning in ways that directly affect clinical decision-making [2,4]. Errors of clinical consequence have been shown to occur more frequently when ad hoc interpreters, including untrained staff or family members, are used compared with professional medical interpreters [2]. Such errors have been associated with increased risk of adverse events, misunderstanding of discharge instructions, reduced adherence to treatment, and decreased patient satisfaction [1]. Moreover, even seemingly minor inaccuracies—such as incorrect medication dosing, failure to convey symptom duration, or omission of qualifiers (e.g., “intermittent” vs. “constant”)—can lead to misdiagnosis, inappropriate testing, or unsafe prescribing. Systematic reviews indicate that professional interpreter services are associated with improved comprehension, safer care processes, and, in some cases, reduced health disparities among LEP populations [1,4]. As healthcare systems increasingly integrate digital and AI-based tools in both written and oral communication, concerns remain regarding accuracy in translation, underscoring the continued importance of clinically trained human interpreters in high-stakes interactions.

While AI voice-assisted technologies hold promise for expanding access to language services, their widespread implementation has outpaced the development of rigorous evidence regarding their safety, accuracy, and impact on communication quality [1,13]. Data suggest that many healthcare organizations have adopted commercial translation platforms without systematic evaluation of their clinical appropriateness or limitations [14,15,16]. Linguistic and clinical translation errors such as those found in our study raise concerns about the extent to which technological solutions are being substituted for, rather than complementing, professionally trained interpreters.

Qualified medical interpreters are trained to convey not only linguistic meaning but also contextual, cultural, and relational aspects of communication. Qualified interpreters receive instruction in medical terminology, ethical principles, and standardized interpretation techniques designed to preserve clinical intent and minimize distortion [34,35]. They are skilled in managing ambiguity, clarifying misunderstandings, and facilitating bidirectional dialogue between patients and providers. Such relational skills remain difficult to replicate through automated systems. Prior research demonstrates that interpreter-mediated encounters are associated with improved patient comprehension, reduced disparities in care, and lower rates of adverse events [1]. Nevertheless, qualified medical interpreters are also not 100% accurate, and clinicians should be encouraged to confirm understanding with their patients through communication methods like teach-back [36].

## 5. Conclusions

In summary, this exploratory study demonstrates that while the Google Translate speech/voice mode may offer a promising avenue for expanding language access in clinical settings, it also may fall short of the accuracy and reliability achieved by qualified in-person medical interpreters. In our study, the significantly higher rates of linguistic and clinical errors using Google Translate —particularly in less commonly spoken languages—highlight potential risks to patient safety and quality of care. These findings underscore the continued need for professional human interpreters to ensure clinically sound communication.

Future research should build on the findings of this study by conducting larger, multi-site investigations that incorporate diverse clinical settings, languages, and patient populations. Longitudinal designs and mixed-method approaches may provide deeper insight into how communication errors affect health outcomes, patient experiences, and healthcare utilization over time [10,37]. Comparative effectiveness studies examining hybrid models that combine AI tools with professional interpreter oversight are also warranted. The ability of AI tools to incorporate pragmatic discourse should also be studied. Ultimately, while technological innovations may enhance access in limited circumstances, they cannot replace the relational, ethical, and contextual competencies of trained human interpreters. A balanced, evidence-based approach that integrates technology as a supportive—not substitutive—resource offers a promising path toward equitable and safe communication for patients with limited English proficiency.

### Limitations

This exploratory study has several limitations that should be considered when interpreting its findings. First, the sample size was relatively small, consisting of 14 recorded clinical encounters across six languages. While the use of authentic, real-world interactions strengthens ecological validity, the limited number of cases restricts the generalizability of the results. The findings may not fully represent the wide range of clinical contexts, provider communication styles, patient characteristics, and linguistic variations present in broader healthcare settings. Larger, multi-site studies are needed to confirm the observed patterns across diverse populations and institutional environments.

Second, the study focused on a selected set of languages, including Amharic, Arabic, Burmese, Spanish, Swahili, and Vietnamese. Although these languages reflect important immigrant and refugee populations in our catchment area, they do not capture the full spectrum of linguistic diversity encountered in healthcare systems. Performance may differ for other commonly spoken or regionally specific languages, dialects, and lesser spoken languages. Additionally, variations in accent, speech rate, and colloquial expression were not systematically analyzed, which may have influenced translation accuracy for both human and automated interpreters.

Third, translation accuracy and clinical significance were assessed through expert review, which necessarily involves a degree of bias. Although standardized criteria were used to evaluate partial and clinically significant errors, translations of clinical impact may vary among reviewers. Differences in professional background and clinical specialty could influence assessments. Further, inter-rater reliability was evaluated on only 12% of the total dataset, which may not represent the reliability across all data points. This should be considered to avoid overinterpretation of the data.

Fourth, this study evaluated only one AI-assisted voice translation application (Google Translate) and did not examine variability across different platforms, software versions, or system updates. Performance may differ substantially among commercial products and may change over time as algorithms are refined. Consequently, the results should not be interpreted as representative of all AI-based interpretation technologies. Similarly, interpreter performance may vary based on certification level, experience, fatigue, and familiarity with specific clinical domains, factors that were not systematically controlled in this analysis.

## Figures and Tables

**Table 1:** Sample of Physician Speech Segments.

Physician Speech Segments
Okay so what I’m going to do now is I’m going to listen to your heart and lungs okay because you’re a new patient and then I really just want to do an exam on your ah muscles okay. The musculoskeletal exam and I want to look at your left leg a little bit closely okay.
it could be ah a cyst which I’ll see if you have any cysts sometimes it’s called a baker’s cyst in the back of your knee which could cause pain ah like I said you got that study, the DVT so that’s good you don’t have any clots and then ah it could be other things like ah of course we want to rule out any signs of infection which I don’t think it is since it’s been happening for 6 months and then just other signs of Edema cause this burning pain usually if you have a burning pain it can be due to swelling and you’re saying it feels swollen so that could explain that and then we could talk about if we need to do any blood work or anything like that and possible treatments as well.
But yeah if your doing fine at home then probably we don’t need to adjust your medications too much. But, uh, again, if at home your blood pressure is running like 160, 170 s, we do need to increase the dose of the lisinopril. As long as we can control your blood pressure, and can control your (.) and control your lipid, that can help to prevent other the more severe = events down the road.
So you know how to recognize the signs of hypoglycemia. Like low blood sugars, say you are sweating a lot, you are having a period of dizziness, those are the signs of low blood sugar and you may need to take a look at your meter and see what is the blood sugar level. If it’s lower than 80, you may want to get something to combat your blood sugar a lit bit. Otherwise, it’s also dangerous. Just like DKA is dangerous, and low blood sugar is also dangerous. Does that make sense?
Okay. Yeah, according to the guideline, because you have type 1 diabetes for more than 5 years is recommended to uh check the microalbumin to see how’s your kidney function annually. If its, say if it’s damaged we can add medication to you to help you to prevent further damage of your kidneys because we know that high blood pressure and diabetes those are two most common causes of the kidney damages in the United States. Have you ever heard about that?

**Table 2:** Coding examples.

Original English	Translated Speech	Linguistic Accuracy and Reason	Is Error Clinically Significant and Reason
it could be ah a cyst which I’ll see if you have any CYSTS sometimes it’s called a baker’s CYST in the back of your knee which could cause pain.	It might be a SWELLING, of which I will check to see if you have any SWELLING. Sometimes it is called a ‘baker’ SWELLING, behind your knee. Which can cause pain.	Partially Wrong—swelling is not the same as cyst.	Not clinically significant. A cyst resembles swelling in that it typically appears as a raised, palpable lump beneath the skin, leading to a localized increase in volume. Both cysts and general swelling may be associated with tenderness, pressure, redness, and warmth—especially when a cyst becomes inflamed or infected.
Every morning even if you don’t have a headache.	If you don’t have a headache every morning.	Totally Wrong: even if you don’t have a headache is the direct opposite of if you don’t have a headache.	Clinically Significant—the 2 sentences are direct opposite of each other and can cause confusion in implementing medication instructions
check to see = If you have any um autoimmune disease. So, when like the body attacks itself, = So to see if there’s like rheumatoid arthritis,	Therefore, to see should do some laboratory tests. To do any self-prevention.	Totally wrong. When the body attacks itself is different than self-prevention. No mention of autoimmune disease.	Clinically Significant—self prevention is proactive and different than self-attack which is destructive.

**Table 3:** Linguistic and Clinical accuracy of APP and QMI Translations by language (*n* = 14 sentences per language per condition).

Language	APP				QMI			
	Linguistically Wrong		Clinically Wrong		Linguistically Wrong		Clinically Wrong	
	*n*	%	*n*	%	*n*	%	*n*	%
Commonly Spoken								
Spanish	3	21.4%	2	14.3%	0	0.0%	0	0.0%
Vietnamese	3	21.4%	2	14.3%	2	14.3%	1	7.1%
Sub-Total	6	21.4%	4	14.3%	2	7.1%	1	3.6%
Less Commonly Spoken								
Amharic	6	42.9%	4	28.6%	9	64.3%	3	21.4%
Arabic	7	50.0%	5	35.7%	0	0.0%	0	0.0%
Burmese	4	28.6%	8	57.1%	3	21.4%	0	0.0%
Swahili	5	35.7%	7	50.0%	3	21.4%	0	0.0%
Sub-Total	22	39.3%	24	42.9%	15	26.8%	3	5.4%
TOTAL	28	33.3%	28	33.3%	17	20.2%	4	4.8%

## Data Availability

The original contributions presented in this study are included in the article. Further inquiries can be directed to the corresponding author.
